# Effect of Cationic Lipid Type in Folate-PEG-Modified Cationic Liposomes on Folate Receptor-Mediated siRNA Transfection in Tumor Cells

**DOI:** 10.3390/pharmaceutics11040181

**Published:** 2019-04-15

**Authors:** Yoshiyuki Hattori, Satono Shimizu, Kei-ichi Ozaki, Hiraku Onishi

**Affiliations:** 1 Department of Drug Delivery Research, Hoshi University, 2-4-41 Ebara, Shinagawa, Tokyo 142-8501, Japan; s.sato1911@gmail.com (S.S.); onishi@hoshi.ac.jp (H.O.); 2 Education and Research Center for Pharmaceutical Sciences, Osaka University of Pharmaceutical Sciences, 4-20-1 Nasahara, Takatsuki, Osaka 569-1094, Japan; kozak@gly.oups.ac.jp

**Keywords:** cationic liposome, folate, folate receptor, cationic cholesterol derivative, siRNA delivery, gene knockdown, tumor-targeting

## Abstract

In this study, we examined the effect of cationic lipid type in folate (FA)-polyethylene glycol (PEG)-modified cationic liposomes on gene-silencing effects in tumor cells using cationic liposomes/siRNA complexes (siRNA lipoplexes). We used three types of cationic cholesterol derivatives, cholesteryl (3-((2-hydroxyethyl)amino)propyl)carbamate hydroiodide (HAPC-Chol), *N*-(2-(2-hydroxyethylamino)ethyl)cholesteryl-3-carboxamide (OH-Chol), and cholesteryl (2-((2-hydroxyethyl)amino)ethyl)carbamate (OH-C-Chol), and we prepared three types of FA-PEG-modified siRNA lipoplexes. The modification of cationic liposomes with 1–2 mol % PEG-lipid abolished the gene-silencing effect in human nasopharyngeal tumor KB cells, which overexpress the FA receptor (FR). In contrast, FA-PEG-modification of cationic liposomes restored gene-silencing activity regardless of the cationic lipid type in cationic liposomes. However, the optimal amount of PEG-lipid and FA-PEG-lipid in cationic liposomes for selective gene silencing and cellular uptake were different among the three types of cationic liposomes. Furthermore, in vitro transfection of polo-like kinase 1 (PLK1) siRNA by FA-PEG-modified liposomes exhibited strong cytotoxicity in KB cells, compared with PEG-modified liposomes; however, in in vivo therapy, intratumoral injection of PEG-modified PLK1 siRNA lipoplexes inhibited tumor growth of KB xenografts, as well as that of FA-PEG-modified PLK1 siRNA lipoplexes. From these results, the optimal formulation of PEG- and FA-PEG-modified liposomes for FR-selective gene silencing might be different between in vitro and in vivo transfection.

## 1. Introduction

RNA interference (RNAi) is a powerful gene-silencing process that holds great promise in the field of cancer therapy. Small double-stranded RNAs, i.e., synthetic small interfering RNAs (siRNAs) suppress the expression of a target gene by triggering specific degradation of the complementary mRNA sequence [1], and therefore siRNA therapeutics have become an increasingly important strategy for anticancer therapy [2]. For siRNA therapy against tumors, siRNAs are designed for targeting mRNAs transcribed from a tumor-causing gene, and siRNAs are introduced into the cytoplasm of tumor cells to cause cleavage of the target mRNA. For example, polo-like kinase 1 (PLK1) is a key regulator for cell mitosis, and its expression is elevated in many types of human tumors [3,4]. The inhibition of PLK1 expression by PLK1 siRNA can lead to death of tumor cells with multiple stages of mitosis [5], and therefore PLK1 is expected to be one of potential targets for siRNA therapy against tumors. However, the success of siRNA therapy relies on the development of safe and efficacious delivery systems that can introduce siRNAs into target tumor cells [2,6,7].

For effective transfection of siRNAs into tumor cells, siRNA carriers such as cationic liposomes are currently the most widely validated means [8]. Many different cationic lipids have been synthesized for lipid-based gene delivery [9] and shown activity in delivering siRNA into cells [10]. For siRNA delivery by cationic liposomes, cationic cholesterol derivatives have often been used [11]. Recently, we reported that cationic liposomes composed of cholesteryl (3-((2-hydroxyethyl)amino)propyl)carbamate hydroiodide (HAPC-Chol), *N*-(2-(2-hydroxyethylamino)ethyl)cholesteryl-3-carboxamide (OH-Chol), or cholesteryl (2-((2-hydroxyethyl)amino)ethyl)carbamate (OH-C-Chol) could efficiently suppress the expression of target genes by siRNA in cells [12,13]. Regarding the liposomal formulation, we previously reported that cationic liposomes composed of OH-Chol/DOPE and OH-C-Chol/DOPE exhibited high gene silencing efficacies compared with cationic nanoparticles composed of OH-Chol/Tween80 and OH-C-Chol/Tween80, respectively [14]. DOPE is thought to improve transfection efficiency by destabilizing the endosomal membrane [15,16], thereby facilitating the release of siRNAs into the cytoplasm. Therefore, a combination of cationic cholesterol derivative with DOPE in cationic liposomes might be a suitable formulation in siRNA delivery. However, selective delivery of siRNAs into tumor cells by cationic liposomes must be achieved for clinical applications.

Folate receptors (FRs) have been found to be overexpressed in a wide range of tumors, including ovary, uterus, lung, kidney, breast, colon, prostate, and brain cancers [17,18,19]. The following four isoforms of FR have been identified: folate receptor alpha (FR-α), beta (FR-β), delta (FR-Δ), and gamma (FR-γ); and FR-α and FR-β are attached to the cell by a GPI-anchor [18]. Among these FR isoforms, FR-α is the most widely studied as a biomarker for tumors, because a few normal tissues have been found to express FR-α, although most express the protein at much lower levels than are detected in FR-α-positive carcinoma. Therefore, FR-mediated tumor targeting has emerged as an attractive method of active targeting of siRNAs into tumor cells by cationic liposomes. When folic acid (FA) or its conjugates bind to FRs, they are taken up into the cells via receptor-mediated endocytosis. Therefore, FA-polyethylene glycol (PEG)-modification has been employed in cationic liposomes to facilitate the uptake of siRNA lipoplexes into tumor cells. Generally, PEGylated cationic liposomes can significantly reduce nonspecific gene transfer. However, conjugation of folate to the PEG chain can restore the cellular association with FR-positive tumors. To the best of our knowledge, there have been no reports about the effect of cationic lipids in FA-PEG-modified cationic liposomes on FR-targeting, although several studies have investigated the application of FA-PEG-modified cationic liposomes for siRNA delivery into FR-expressing cells [20,21,22,23]. In this study, to examine the effect of cationic lipid type in FA-PEG-modified cationic liposomes on gene-silencing effects, we selected three kinds of cationic cholesterol derivatives, HAPC-Chol, OH-Chol, and OH-C-Chol, and prepared three types of FA-PEG-modified cationic liposomes composed of cationic cholesterol derivatives and DOPE for the evaluation of gene-silencing effects. Here, we found that in FR-selective siRNA delivery, the cationic lipid type in FA-PEG-modified cationic liposomes affected an optimal amount of FA-PEG_2000_-DSPE in liposomal formulation, and the optimized formulation of FA-PEG-modified cationic liposomes by in vitro transfection was not necessarily correlated with formulation by in vivo transfection.

## 2. Materials and Methods

### 2.1. Materials

*N*-(2-(2-Hydroxyethylamino)ethyl)cholesteryl-3-carboxamide (OH-Chol) and cholesteryl (2-((2-hydroxyethyl)amino)ethyl)carbamate (OH-C-Chol) were synthesized as described previously [14,24,25]. Cholesteryl (3-((2-hydroxyethyl)amino)propyl)carbamate hydroiodide (HAPC-Chol) was synthesized as described previously [26]. Methoxy-poly(ethyleneglycol)-distearylphosphatidylethanolamine (PEG_2000_-DSPE/SUNBRIGHT DSPE-020CN, and PEG_5000_-DSPE/SUNBRIGHT DSPE-050CN, and PEG mean molecular weight, 2000 and 5000, respectively) and 1,2-dioleoyl-*sn*-glycero-3-phosphoethanolamine (DOPE, COATSOME ME-8181) were obtained from NOF Co. Ltd. (Tokyo, Japan). All other chemicals were of the highest grade available.

### 2.2. Small Interfering RNAs

The siRNAs targeting nucleotides of firefly pGL4 luciferase (Luc), enhanced green fluorescent protein (EGFP), human polo-like kinase 1 (PLK1), and non-silencing siRNA control (Cont) as a negative control were synthesized by Sigma Genosys (Tokyo, Japan). Alexa Fluor^®^488-labeled AllStars Negative Control siRNA (AF-siRNA) was obtained from Qiagen (Valencia, CA, USA). The siRNA sequences for Luc siRNA: sense strand: 5′-GGACGAGGACGAGCACUUCUU-3′ and antisense strand: 5′-GAAGUGCUCGUCCUCGUCCUU-3. The siRNA sequences for EGFP siRNA: sense strand: 5′-GGCUACGUCCAGGAGCGCACCTT-3′ and antisense strand: 5′-GGUGCGCUCCUGGACGUAGCCTT-3. The siRNA sequences of the PLK1 siRNA were as reported previously [27]. The siRNA sequences of the Cont siRNA were as reported previously [28].

### 2.3. Preparation of Cationic Liposomes and siRNA Lipoplexes

FA-PEG-distearoylphosphatidylethanolamine (FA-PEG-DSPE) (mean MW of PEG: 2000 and 5000) was synthesized as described previously [29]. Cationic liposomes were prepared from HAPC-Chol/DOPE (composition designated as LP-HAPC), OH-Chol/DOPE (composition designated as LP-OH), and OH-C-Chol/DOPE (composition designated as LP-OH-C), at a molar ratio of 3:2. PEG_2000_- and FA-PEG_2000_-modified cationic liposomes were incorporated at 1, 2, or 3 mol% PEG_2000_-DSPE and FA-PEG_2000_-DSPE, respectively, into the cationic liposomal formulation, as shown in Table 1. PEG_5000_- and FA-PEG_5000_-modified LP-HAPC (LP-HAPC-PEG_5000_ and LP-HAPC-FA-PEG_5000_) were incorporated with 1 mol % PEG_5000_-DSPE and FA-PEG_5000_-DSPE, respectively, into the formulation of LP-HAPC.

For the preparation of cationic liposomes by a thin-film hydration method, cationic cholesterol derivative, DOPE, and PEG-DSPE or FA-PEG-DSPE were dissolved in chloroform, and then the chloroform was evaporated under vacuum on a rotary evaporator at 60 °C to obtain a thin film. The thin film was hydrated with water at 60 °C by vortex mixing. The liposomes were sonicated in a bath-type sonicator (Bransonic^®^ 2510J-MTH, 100W, Branson UL Trasonics Co., Danbury, CT, USA) to produce liposomes with a final size of approximately 100 nm.

In in vitro transfection, to prepare cationic liposome/siRNA complexes (siRNA lipoplexes), each cationic liposome was added to 50 pmol siRNA at a charge ratio (+:−) of 7:1 with vortex mixing for 10 s and left at room temperature for 15 min. The charge ratio (+:−) of cationic liposome:siRNA is expressed as the molar ratio of cationic lipid to siRNA phosphate.

### 2.4. Size and ζ-Potential of Cationic Liposomes and siRNA Lipoplexes

The siRNA lipoplexes were formed by addition of cationic liposomes to 5 μg Cont siRNA at a charge ratio (+:−) of 7:1 with vortex mixing for 10 s. The particle size distributions of cationic liposomes and siRNA lipoplexes were measured by the cumulant method using a light-scattering photometer (ELS-Z2, Otsuka Electronics Co., Ltd., Osaka, Japan) at 25 °C after diluting the dispersion to an appropriate volume (~1.5 mL) with water. The ζ-potentials of cationic liposomes and siRNA lipoplexes were measured by electrophoresis light-scattering methods using ELS-Z2 at 25 °C after diluting the dispersion with an appropriate volume (~1.5 mL) with water.

### 2.5. Cell Culture

Human nasopharyngeal tumor KB cells (also known as a subline of human cervix adenocarcinoma HeLa) were obtained from the Cell Resource Center for Biomedical Research, Tohoku University (Miyagi, Japan). KB cells were cultured in RPMI-1640 medium with 10% heat-inactivated fetal bovine serum (FBS) and 100 μg/mL kanamycin in a humidified atmosphere containing 5% CO_2_ at 37 °C.

For the preparation of KB cells stably expressing firefly pGL4 luciferase, KB cells were plated on 35-mm culture dishes. Twenty-four hours later, the cells were transfected with 2 μg of plasmid DNA encoding firefly pGL4 luciferase under the control of a cytomegalovirus (CMV) promoter (pGL4.51[luc2/CMV/Neo] Vector, Promega, Madison, WI, USA) using Lipofectamine 2000 transfection reagent (Invitrogen, Thermo Fisher Scientific, Inc., Carlsbad, CA, USA). The transfected cells were selected in medium with 1200 μg/mL G418 sulfate. G418-resistant colonies were subcultured and established as a permanent cell line expressing firefly pGL4 luciferase (KB-Luc).

For the preparation of KB cells stably expressing EGFP, KB cells were transfected with 2 μg of plasmid DNA encoding EGFP under the control of a CMV promoter (pEGFP-C1, Clontech, Palo Alto, CA, USA) using Lipofectamine 2000 transfection reagent. The transfected cells were selected in medium with 1 mg/mL G418 sulfate. G418-resistant colonies were subcultured and established as a permanent cell line expressing EGFP (KB-EGFP).

### 2.6. Gene Silencing Effect by FA-PEG-Modified siRNA Lipoplexes in KB-Luc Cells

The KB-Luc cells were seeded in 6-well culture plate at a density of 3 × 10^5^ cells per well, 24 h prior to transfection. The siRNA lipoplexes were formed by the addition of cationic liposomes into 50 pmol Cont siRNA or Luc siRNA with vortex mixing for 10 s and left at room temperature for 15 min. For siRNA transfection, each siRNA lipoplex was diluted in 1 mL of folate-deficient RPMI-1640 medium (Invitrogen) supplemented with 10% FBS and then the mixture was added to the cells (final 50 nM siRNA concentration). Lipofectamine RNAiMAX lipoplexes (Invitrogen) were prepared in accordance with the manufacturer’s protocol and then added to the cells. Forty-eight hours after the transfection, luciferase activity was measured as counts per sec (cps)/μg protein using the luciferase assay system (PicaGene MelioraStar-LT Luminescence Reagent, Toyo Ink Mfg. Co. Ltd., Tokyo, Japan) and BCA reagent (Pierce™ BCA Protein Assay Kit, Pierce, Rockford, IL, USA). Luciferase activity (%) was calculated as relative to the luciferase activity (cps/μg protein) of untransfected cells.

### 2.7. Gene Silencing Effect by FA-PEG-Modified siRNA Lipoplexes in KB-EGFP Cells

The KB-EGFP cells were seeded in 6-well culture plate at a density of 3 × 10^5^ cells per well, 24 h prior to transfection. The siRNA lipoplexes were formed by addition of cationic liposomes into 50 pmol Cont siRNA or EGFP siRNA with vortex mixing for 10 s and left at room temperature for 15 min. For siRNA transfection, each siRNA lipoplex was diluted in 1 mL of folate-deficient RPMI-1640 medium supplemented with 10% FBS and then the mixture was added to the cells (final 50 nM siRNA concentration). Lipofectamine RNAiMAX lipoplexes were prepared in accordance with the manufacturer’s protocol and then added to the cells. Forty-eight hours after transfection, EGFP expression levels in the cells were determined by examining fluorescence intensity using a FACSVerse^TM^ flow cytometer (Becton Dickinson, San Jose, CA, USA) equipped with a 488-nm argon ion laser. Data for 10,000 fluorescence events were obtained by recording forward scatter (FSC), side scatter (SSC), and green (530/30 nm) fluorescence. EGFP expression level (%) was calculated as relative to the fluorescence intensity of untransfected cells.

### 2.8. Cytotoxicity by FA-PEG-Modified siRNA Lipoplexes

The KB cells were seeded in 96-well culture plate, 24 h prior to transfection. Each siRNA lipoplex with 50 pmol Cont siRNA was diluted in 1 mL of folate-deficient RPMI-1640 medium supplemented with 10% FBS, and then the mixture (100 μL) was added to the cells at 50% confluency in the well (final 50 nM siRNA concentration). After a 24 h incubation period, cell numbers were determined using a WST-8 assay (Cell Counting Kit-8, Dojindo Laboratories, Kumamoto, Japan). Cell viability was expressed as relative to the absorbance at 450 nm of untransfected cells.

### 2.9. Cellular Association with FA-PEG-Modified siRNA Lipoplexes

The KB cells were seeded in 6-well culture plate at a density of 3 × 10^5^ cells per well, 24 h prior to transfection. Each siRNA lipoplex with 50 pmol AF-siRNA was diluted in 1 mL of folate-deficient RPMI-1640 medium (50 nM siRNA) supplemented with 10% FBS and then added to the cells. After 3 h incubation, the cells were washed twice with 1 mL phosphate-buffered saline (PBS) to remove any unbound lipoplexes. The amount of AF-siRNA in the cells was determined by examining fluorescence intensity using a FACSVerse^TM^ flow cytometer equipped with a 488-nm argon ion laser. Data for 10,000 fluorescence events were obtained by recording forward scatter (FSC), side scatter (SSC), and green (530/30 nm) fluorescence.

### 2.10. Gel Retardation Assay

One microgram of siRNAs was mixed with cationic liposomes at various charge ratios (+:−) from 1:1 to 4:1. After 15 min incubation of the siRNA lipoplexes, they were analyzed by 18% acrylamide gel electrophoresis for siRNA in Tris-Borate-EDTA buffer (pH 8.0) and detected by ethidium bromide staining, as reported previously [30].

### 2.11. Accessibility of siRNA in siRNA Lipoplexes

The siRNA association by cationic liposomes was analyzed by exclusion assay using an SYBR^®^ Green I Nucleic Acid Gel Stain (Takara Bio Inc., Shiga, Japan). The siRNA lipoplexes were formed at charge ratios (+:−) of 1:1, 2:1, 3:1, and 4:1. The siRNA lipoplexes with 1 μg of siRNA in a volume of 100 μL of Tris-HCl buffer (pH 8.0) were mixed with 100 μL of 2500-fold diluted SYBR^®^ Green I Nucleic Acid Gel Stain solution with Tris-HCl buffer, and then incubated for 30 min. The fluorescence was measured at an emission wavelength of 535 nm with an excitation wavelength of 485 nm using a fluorescence plate reader (ARVO X2, Perkin Elmer, Waltham, MA, USA). As a control, the value of fluorescence obtained upon addition of free siRNA solution was set as 100%. The amount of siRNA available to interact with the SYBR^®^ Green I was expressed as a percentage of the control.

### 2.12. Antiproliferative Activity

The KB cells were seeded in 96-well culture plate, 24 h prior to transfection. Each siRNA lipoplex with 50 pmol Cont siRNA or PLK1 siRNA was diluted in 1 mL of folate-deficient RPMI-1640 medium supplemented with 10% FBS, and then the mixture (100 μL) was added to the cells at 20–30% confluency in the well (final 50 nM siRNA concentration). After the 48 h incubation period, cell viability was measured by a WST-8 assay as described above.

### 2.13. Measurement of Expression Level of PLK1 mRNA

For the knockdown of PLK1 mRNA by transfection with PLK1 siRNA, KB cells were plated into 6-well culture plate at a density of 3 × 10^5^ cells/well. The siRNA lipoplexes with 50 pmol Cont siRNA or PLK1 siRNA were diluted in 1 mL of folate-deficient RPMI-1640 medium (50 nM siRNA) supplemented with 10% FBS and then added to the cells. At 24 h after transfection, total RNA was isolated using NucleoSpin RNA (Macherey-Nagel, GmbH, Düren, Germany). First strand cDNA was synthesized from 1 μg of total RNA using PrimeScript RTase (Takara Bio, Inc., Otsu, Japan). Quantitative (q)PCR was performed using a Roche Light Cycler 96 system (Roche Diagnostics, Basel, Switzerland) and TaqMan Gene expression assays (PLK1, Hs00983227_m1 and GAPDH, Hs02786624_g1, Applied Biosystems, Thermo Fisher Scientific Inc.). Samples were run in triplicate, and the expression levels of PLK1 mRNA were normalized by the amount of GAPDH mRNA in the same sample, and analyzed using the comparative Cq (2^−ΔΔCq^) method [31].

### 2.14. In Vivo Anti-Tumor Effect

All animal experiments were conducted in accordance with the “Guide for the Care and Use of Laboratory Animals” published by the U.S. National Institutes of Health and the “Guide for the Care and Use of Laboratory Animals” adopted by the Institutional Animal Care and the Use Committee of Hoshi University (Tokyo, Japan) (which is accredited by the Ministry of Education, Culture, Sports, Science, and Technology, Japan). Ethical approval for this study was obtained from the Institutional Animal Care and the Use Committee of Hoshi University (permission number: 30-074).

To generate KB tumor xenografts, 1 × 10^7^ cells suspended in 50 μL of PBS were inoculated subcutaneously in the flank region of female BALB/c nu/nu mice (8 weeks of age, CLEA Japan Inc., Tokyo, Japan). The tumor volume was calculated using the formula, tumor volume = 0.5 × a × b^2^, where a and b are the larger and smaller diameters, respectively. When the average volume of the xenograft tumors reached 80 mm^3^ (day 0), LP-HAPC-2mol%PEG_2000_ or LP-HAPC-2mol%FA-PEG_2000_ lipoplexes of 10 μg of Cont siRNA or PLK1 siRNA per tumor were directly injected into xenografts on days 0, 2, and 4. Tumor volume was measured on days 0, 2, 4, 6, and 8. Tumor volume (%) was calculated as relative to each tumor volume at the day 0. At day 8, mice were sacrificed by cervical dislocation, and then the excised tumors were weighed.

### 2.15. Statistical Analysis

The statistical significance of differences between mean values was determined using Student’s *t*-test. A *p*-value of 0.05 or less was considered significant.

## 3. Results

### 3.1. Characterization of FA-PEG-Modified Cationic Liposomes and siRNA Lipoplexes

**Figure 1:**
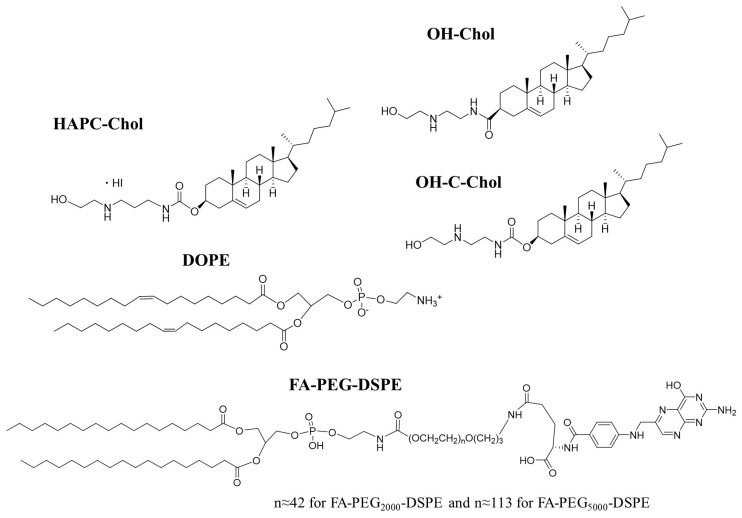
Structure of cationic cholesterol derivatives, neutral helper lipid, and FA-PEG-DSPE: HAPC-Chol; cholesteryl (3-((2-hydroxyethyl)amino)propyl)carbamate hydroiodide, OH-Chol; *N*-(2-(2-hydroxyethylamino)ethyl)cholesteryl-3-carboxamide, OH-C-Chol; cholesteryl (2-((2-hydroxyethyl)amino)ethyl)carbamate, DOPE; 1,2-dioleoyl-*sn*-glycero-3-phosphoethanolamine, FA-PEG-DSPE; FA-PEG-distearoylphosphatidylethanolamine.

Firstly, we examined whether the cationic lipid type in FA-PEG-modified cationic liposomes affected gene silencing after transfection of FA-PEG-modified siRNA lipoplexes into KB cells, which overexpressed FR-α [32]. Here, we used HAPC-Chol, OH-Chol, and OH-C-Chol (Figure 1) as cationic cholesterol derivatives for preparation of cationic liposomes. LP-HAPC, LP-OH, and LP-OH-C were prepared from HAPC-Chol/DOPE, OH-Chol/DOPE, and OH-C-Chol/DOPE, respectively, at a molar ratio of 3:2 (Table 1). For PEGylated cationic liposomes, 1, 2, or 3 mol% PEG_2000_-DSPE was added to the formulation of LP-HAPC, LP-OH, and LP-OH-C (LP-HAPC-1–3mol%PEG_2000_, LP-OH-1–3mol%PEG_2000_, and LP-OH-C-1–3mol%PEG_2000_, respectively). For FR-targeted cationic liposomes, 1, 2, or 3 mol% FA-PEG_2000_-DSPE was added to the formulation of LP-HAPC, LP-OH, and LP-OH-C (LP-HAPC-1–3mol%FA-PEG_2000_, LP-OH-1–3mol%FA-PEG_2000_, and LP-OH-C-1–3mol%FA-PEG_2000_, respectively). In addition, as PEG- or FA-PEG-modified cationic liposomes with a longer PEG chain, 1 mol% PEG_5000_-DSPE and FA-PEG_5000_-DSPE, respectively, were added to the formulation of LP-HAPC (LP-HAPC-1mol%PEG_5000_, and LP-HAPC-1mol%FA-PEG_5000_, respectively).

In HAPC-Chol-based liposomes, the sizes of LP-HAPC, LP-HAPC-1–3mol%PEG_2000_, LP-HAPC-1–3mol%FA-PEG_2000_, LP-HAPC-1mol%PEG_5000_, and LP-HAPC-1mol%FA-PEG_5000_ were approximately 90–110 nm, polydispersity index (PDI) 0.21–0.26, and the ζ-potentials were approximately +35–47 mV (Table 2). In OH-Chol-based liposomes, the sizes of LP-OH, LP-OH-1–3mol%PEG_2000_, and LP-OH-1–3mol%FA-PEG_2000_ were approximately 84–110 nm (PDI 0.17–0.31), and the ζ-potentials were approximately +34–47 mV (Table 2). In OH-C-Chol-based liposomes, the sizes of LP-OH-C, LP-OH-C-1–3mol%PEG_2000_ and LP-OH-C-1–3mol%FA-PEG_2000_ were approximately 90–110 nm (PDI 0.12–0.26), and the ζ-potentials were approximately +43–52 mV (Table 2).

For the preparation of siRNA lipoplexes, their liposomes were mixed with siRNA at a charge ratio (+:−) of 7:1 as reported previously [13], because siRNA lipoplexes at this charge ratio (+:−) exhibited high gene silencing efficacy (Supplemental Figure S1). In HAPC-Chol-based lipoplexes, the sizes of LP-HAPC, LP-HAPC-1–3mol%PEG_2000_, LP-HAPC-1–3mol%FA-PEG_2000_, LP-HAPC-1mol%PEG_5000_, and LP-HAPC-1mol%FA-PEG_5000_ lipoplexes were approximately 170–210 nm (PDI 0.24–0.28), and the ζ-potentials were approximately 21–40 mV (Table 2). In OH-Chol-based lipoplexes, the sizes of LP-OH, LP-OH-1–3mol%PEG_2000_, and LP-OH-1–3mol%FA-PEG_2000_ lipoplexes were approximately 170–300 nm (PDI 0.22–0.28), and the ζ-potentials were approximately 21–45 mV (Table 2). In OH-C-Chol-based lipoplexes, the sizes of LP-OH-C, LP-OH-C-1–3mol%PEG_2000_, and LP-OH-C-1–3mol%FA-PEG_2000_ lipoplexes were approximately 170–420 nm (PDI 0.12–0.26), and the ζ-potentials were approximately 22–39 mV (Table 2). Regardless the cationic lipid type in cationic liposomes, PEG-modification, or FA-PEG-modification of cationic liposomes trended to decrease the ζ-potentials of cationic liposomes and siRNA lipoplexes. However, the type of cationic cholesterol derivatives in cationic liposomes did not largely affect size and ζ-potential of cationic liposomes and siRNA lipoplexes.

### 3.2. Effect of Cationic Lipid of FA-PEG-Modified Cationic Liposomes on In Vitro Gene Knockdown Efficacy

**Figure 2:**
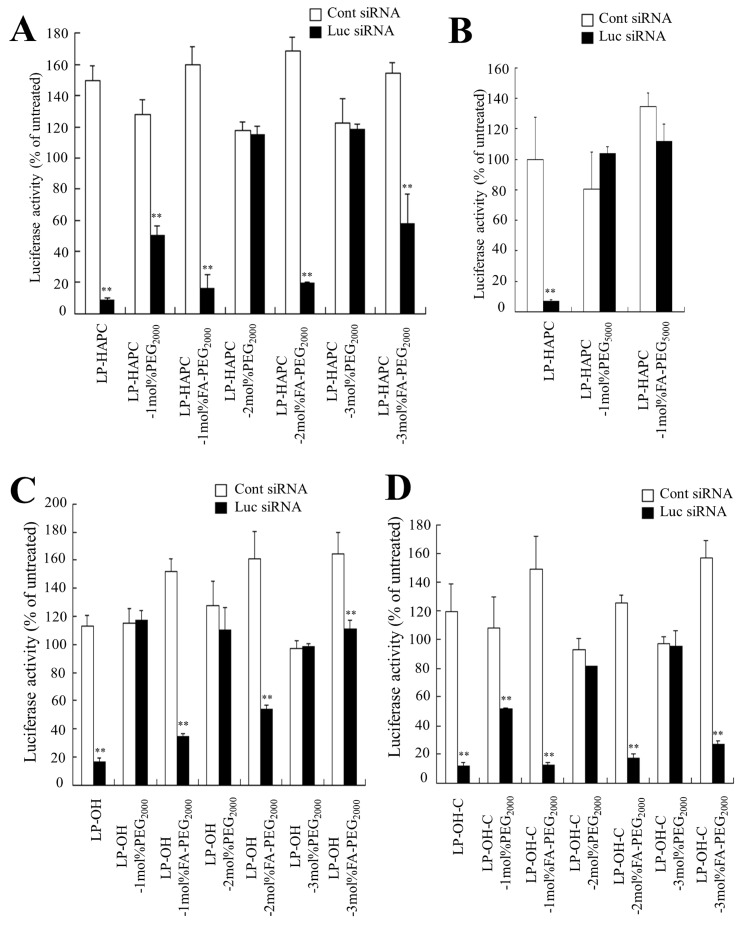
Effect of FA-PEG modification of cationic liposomes on suppression of luciferase expression in KB-Luc cells after transfection with FA-PEG-modified siRNA lipoplexes. (**A**) LP-HAPC-1–3mol%PEG_2000_ and LP-HAPC-1–3mol%FA-PEG_2000_, (**B**) LP-HAPC-1mol%PEG_5000_ and LP-HAPC-1mol%FA-PEG_5000_, (**C**) LP-OH-1–3mol%PEG_2000_ and LP-OH-1–3mol%FA-PEG_2000_, (**D**) LP-OH-C-1–3mol%PEG_2000_ and LP-OH-C-1–3mol%FA-PEG_2000_ were used. The siRNA lipoplexes with Cont siRNA or Luc siRNA were added to KB-Luc cells at 50 nM siRNA, and the luciferase assay was carried out 48 h after incubation. Each column represents the mean + SD (*n* = 3). ** *p* < 0.01, compared with Cont siRNA.

To examine the gene silencing effect in FR-expressing cells by FA-PEG-modified siRNA lipoplexes, KB-Luc cells were incubated with siRNA lipoplexes modified with 1–3 mol% PEG_2000_-DSPE or FA-PEG_2000_-DSPE, and the gene silencing activity was assessed by assaying luciferase activity. LP-HAPC, LP-OH, and LP-OH-C lipoplexes with Luc siRNA strongly suppressed luciferase activity (>80% knockdown, compared with Cont siRNA) (Figure 2A,C,D). In HAPC-Chol- and OH-C-Chol-based liposomes, above 2 mol% PEG-modification of LP-HAPC and LP-OH-C lipoplexes with PEG_2000_-DSPE completely abolished the gene silencing effect; however, LP-HAPC-2mol%FA-PEG_2000_ and LP-OH-C-2mol%FA-PEG_2000_ lipoplexes with Luc siRNA exhibited strong suppression of luciferase activity (Figure 2A,D). In contrast, in OH-Chol-based liposomes, above 1 mol% PEG-modification of LP-OH lipoplexes with PEG_2000_-DSPE abolished the gene silencing effect; however, LP-OH-1mol%FA-PEG_2000_ lipoplexes with Luc siRNA exhibited strong suppression of luciferase activity (Figure 2C). From these results, in FR-mediated gene silencing, the optimal amount of PEG_2000_-DSPE or FA-PEG_2000_-DSPE in the liposomal formulation may be affected by the cationic lipid type in FA-PEG-modified liposomes. Regarding the length of the PEG chain between FA and lipid, both LP-HAPC-1mol%PEG_5000_ and LP-HAPC-1mol%FA-PEG_5000_ lipoplexes did not exhibit gene silencing activity (Figure 2B), indicating that a longer PEG chain inhibited FR-mediated uptake by cells. From these results, optimal modifications of PEG_2000_-DSPE or FA-PEG_2000_-DSPE in formulations of LP-HAPC, LP-OH, and LP-OH-C were 2 mol%, 1 mol%, and 2 mol%, respectively, for selective FR-mediated gene silencing in tumor cells.

### 3.3. Cytotoxicity by FA-PEG-Modified siRNA Lipoplexes

**Figure 3:**
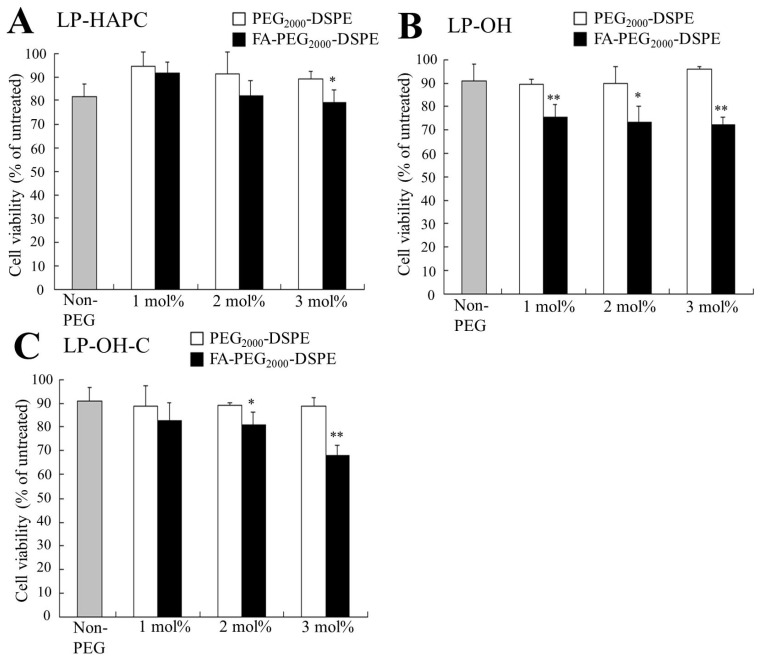
Effect of FA-PEG-modification of cationic liposomes on cell viability 24 h after transfection with FA-PEG-modified siRNA lipoplexes into KB-Luc cells. (**A**) LP-HAPC-1–3mol%PEG_2000_ and LP-HAPC-1–3mol%FA-PEG_2000_, (**B**) LP-OH-1–3mol%PEG_2000_ and LP-OH-1–3mol%FA-PEG_2000_, (**C**) LP-OH-C-1–3mol%PEG_2000_ and LP-OH-C-1–3mol%FA-PEG_2000_ were used. The siRNA lipoplexes were added to KB cells at 50 nM siRNA. Each column represents the mean + SD (*n* = 4). Each column represents the mean + SD (*n* = 3). * *p* < 0.05, ** *p* < 0.01, compared with PEG_2000_-DSPE.

To examine whether FA-PEG-modification of cationic liposomes affected the cytotoxicity, we investigated cell viability at 24 h after transfection into KB cells with FA-PEG-modified siRNA lipoplexes. LP-HAPC, LP-OH, and LP-OH-C lipoplexes did not exhibit marked cytotoxicity (80–90% cell viability), and PEG-modification of their lipoplexes also did not affect cytotoxicity (approximately 90% cell viability) (Figure 3). However, FA-PEG-modification of LP-HAPC, LP-OH, and LP-OH-C slightly increased cytotoxicity (70–80% cell viability) with increasing the amounts of FA-PEG_2000_-DSPE in the cationic liposomes, compared with the PEG-modification. These results indicated that FA-PEG-modification of cationic liposomes might increase cytotoxicity by increasing the cellular uptake of siRNA lipoplexes.

### 3.4. Association of FA-PEG-Modified siRNA Lipoplexes with Cells

**Figure 4:**
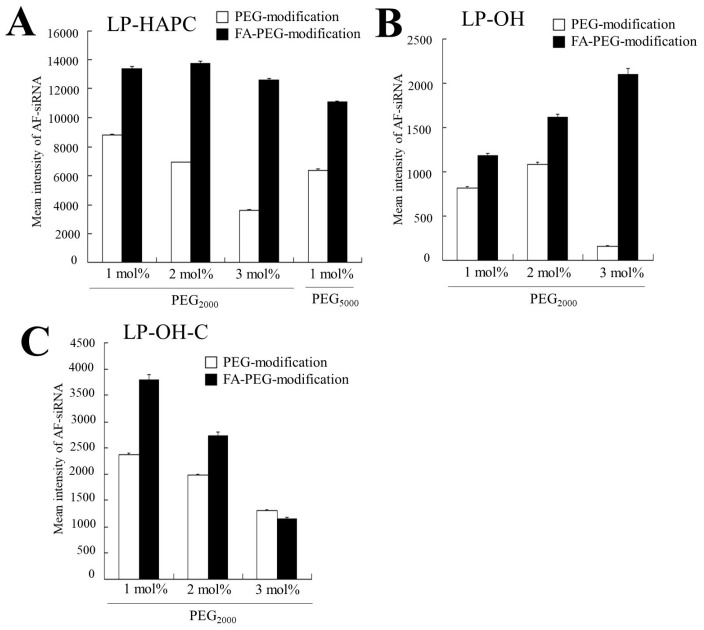
Effect of FA-PEG-modification of cationic liposomes on cellular association at 3 h after transfection of FA-PEG-modified siRNA lipoplexes. (**A**) LP-HAPC-1–3mol%PEG_2000_ and LP-HAPC-1–3mol%FA-PEG_2000_, (**B**) LP-OH-1–3mol%PEG_2000_ and LP-OH-1–3mol%FA-PEG_2000_, (**C**) LP-OH-C-1–3mol%PEG_2000_ and LP-OH-C-1–3mol%FA-PEG_2000_ were used. The siRNA lipoplexes were formed by mixing cationic liposomes with AF-siRNA, and they were added to KB cells at a final concentration of 50 nM siRNA. The association of siRNA lipoplexes with the cells was determined on the basis of Alexa Fluor^®^488-fluorescence by flow cytometry. Each column represents the mean fluorescent intensity + SD (*n* = 3).

To examine the effect of cationic lipid type on cellular association with FA-PEG-modified siRNA lipoplexes, we measured the siRNA amount taken up by KB cells at 3 h after transfection with FA-PEG-modified siRNA lipoplexes (Figure 4). In LP-HAPC, LP-OH, and LP-OH-C, the amount of siRNA in the cells decreased with increasing PEG-modification (Figure 4A–C). In contrast, 1–3 mol% FA-PEG-modification of LP-HAPC and LP-OH exhibited high cellular uptake of siRNA in the cells, compared with PEG-modified ones (Figure 4A,B). However, in LP-OH-C, with an increase of FA-PEG-modification, the amount of siRNA in the cells was decreased substantially, and LP-OH-C-3mol%FA-PEG_2000_ lipoplexes did not enhance cellular association compared with LP-OH-C-3mol%PEG_2000_ lipoplexes (Figure 4C). These results indicated that the cellular association of FA-PEG-modified siRNA lipoplexes might be strongly affected by cationic lipid type in cationic liposomes.

### 3.5. Association of FA-PEG-Modified Cationic Liposomes with siRNA

**Figure 5:**
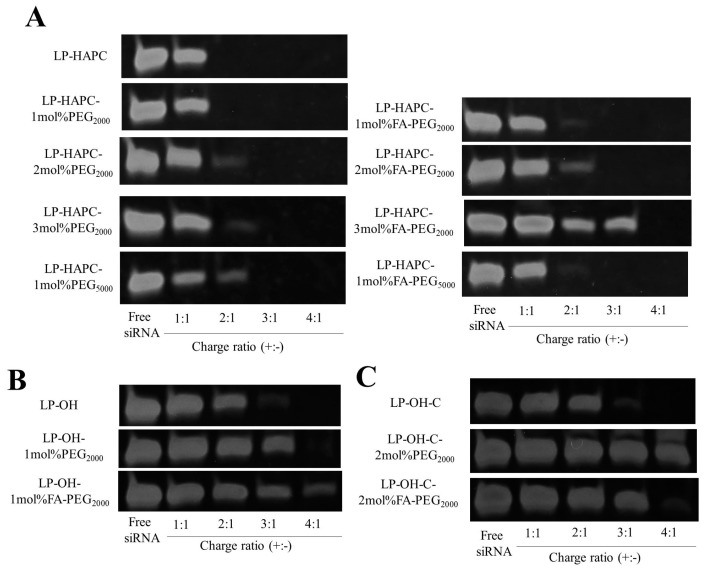
Effect of FA-PEG-modification of cationic liposomes on association of siRNA with FA-PEG-modified cationic liposomes. siRNA association by cationic liposomes was analyzed by gel retardation assay. (**A**) LP-HAPC-1–3mol%PEG_2000_, LP-HAPC-1mol%PEG_5000_, LP-HAPC-1–3mol%FA-PEG_2000_, and LP-HAPC-1mol%FA-PEG_5000_, (**B**) LP-OH-1–3mol%PEG_2000_ and LP-OH-1–3mol%FA-PEG_2000_, (**C**) LP-OH-C-1–3mol%PEG_2000_ and LP-OH-C-1–3mol%FA-PEG_2000_ were used. Each liposome was formed with siRNA at various charge ratios (+:−) from 1:1 to 4:1, and were analyzed using 18% acrylamide gel electrophoresis.

Next, we evaluated the association of FA-PEG-modified cationic liposomes with siRNA. The association of siRNA with each cationic liposome was monitored by gel retardation electrophoresis (Figure 5). The migration pattern of siRNA in siRNA lipoplexes was changed when the siRNA was mixed with cationic liposomes at charge ratios (+:−) from 1:1 to 4:1, and the migration of siRNAs ceased gradually as the charge ratio (+:−) increased. In HAPC-Chol-based liposomes, no migration was observed beyond charge ratios (+:−) of 2:1 in LP-HAPC, LP-HAPC-1mol%PEG_2000_, and LP-HAPC-1mol%FA-PEG_2000_ lipoplexes, of 3:1 in LP-HAPC-2mol%PEG_2000_, LP-HAPC-2mol%FA-PEG_2000_, and LP-HAPC-3mol%PEG_2000_, and of 4:1 in LP-HAPC-3mol%FA-PEG_2000_, (Figure 5A).

This result suggested that the association of siRNA with the cationic liposomes was inhibited with increasing amounts of FA-PEG_2000_-DSPE or PEG_2000_-DSPE. In siRNA transfection, we used a charge ratio (+:−) of 7:1 for the preparation of siRNA lipoplexes; therefore, siRNAs were completely bound to LP-HAPC regardless the PEG- or FA-PEG-modification. In addition, in LP-HAPC-1mol%PEG_5000_ and LP-HAPC-1mol%FA-PEG_5000_ lipoplexes, no migration was observed beyond charge ratios (+:−) of 2:1 (Figure 5A), indicating that the decrease in gene silencing activity in LP-HAPC-1mol%FA-PEG_5000_ lipoplexes (Figure 2B) was not caused by a decrease in the association of cationic liposomes with siRNA by the long PEG chain. Furthermore, LP-OH- and LP-OH-C-based liposomes, beyond charge ratios (+:−) of 4:1 in LP-OH, LP-OH-1mol%PEG_2000_, LP-OH-1mol%FA-PEG_2000_, LP-OH-C, LP-OH-C-2mol%PEG_2000_, LP-OH-C-2mol%FA-PEG_2000_ lipoplexes, no migration or decreased migration was observed (Figure 5B,C). These results indicated that OH-Chol- and OH-C-Chol-based liposomes might make a weaker association with siRNA than HAPC-Chol-based liposomes.

**Figure 6:**
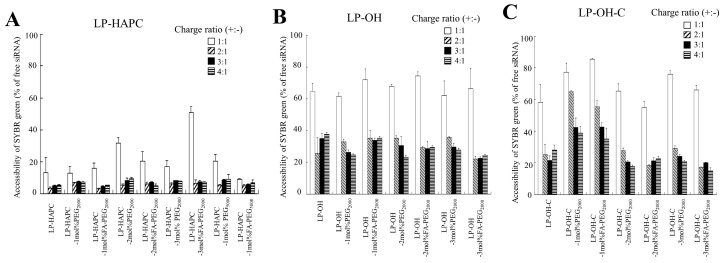
Effect of FA-PEG-modification of cationic liposomes on association of siRNA with FA-PEG-modified cationic liposome. siRNA association by cationic liposomes was analyzed by exclusion assay using an SYBR^®^ Green I Nucleic Acid Gel Stain. (**A**) LP-HAPC-1–3mol%PEG_2000_, LP-HAPC-1mol%PEG_5000_, LP-HAPC-1–3mol%FA-PEG_2000_, and LP-HAPC-1mol%FA-PEG_5000_, (**B**) LP-OH-1–3mol%PEG_2000_ and LP-OH-1–3mol%FA-PEG_2000_, (**C**) LP-OH-C-1–3mol%PEG_2000_ and LP-OH-C-1–3mol%FA-PEG_2000_ were used. The siRNA lipoplexes were formed at various charge ratios (+:−) from 1:1 to 4:1. As a control, the value of fluorescence obtained upon addition of free siRNA solution was set as 100%. The amount of siRNA available to interact with the SYBR^®^ Green I was expressed as a percentage of the control. Each column represents the mean + SD (*n* = 3).

Furthermore, we examined the association of siRNA with each cationic liposome using an exclusion assay with SYBR^®^ Green I. SYBR^®^ Green I is a DNA/RNA-intercalating agent whose fluorescence was dramatically enhanced upon binding to unbound siRNA in cationic lipoplexes. As a result, in all the cationic liposomes, the fluorescence of SYBR^®^ Green I was markedly decreased by the addition of cationic liposomes into the siRNA solution beyond charge ratios (+:−) of 2:1 or 3:1, compared with that in siRNA solution (Figure 6A–C). This result suggested that siRNAs were completely bound to each cationic liposome regardless of PEG or FA-PEG modification of the cationic liposomes. Although a discrepancy between the results from the accessibility of SYBR^®^ Green I (Figure 6) and gel retardation electrophoresis (Figure 5) was observed, siRNAs might be released from siRNA lipoplexes by electrophoresis due to the weak association between siRNA and the cationic liposomes. From the result shown in Figure 6, for all the cationic liposomes used in this study, siRNAs might be completely bound to the cationic liposomes when mixed beyond a charge ratio (+:−) of 3:1.

### 3.6. Suppression of EGFP Expression by FA-PEG-Modified siRNA Lipoplexes

To examine the effect of FA-PEG-modification in cationic liposomes on gene knockdown using FA-PEG-modified siRNA lipoplexes, KB-EGFP cells were incubated with siRNA lipoplexes, and then the gene silencing effect was assessed by assaying the fluorescence intensity in the cells. Here, we decided to use LP-HAPC-2mol%FA-PEG_2000_, LP-OH-1mol%FA-PEG_2000_, and LP-OH-C-2mol%FA-PEG_2000_ as optimal FA-PEG-modified liposomes. In addition, we used Lipofectamine RNAiMax as a commercially available in vitro transfection reagent for siRNAs.

**Figure 7:**
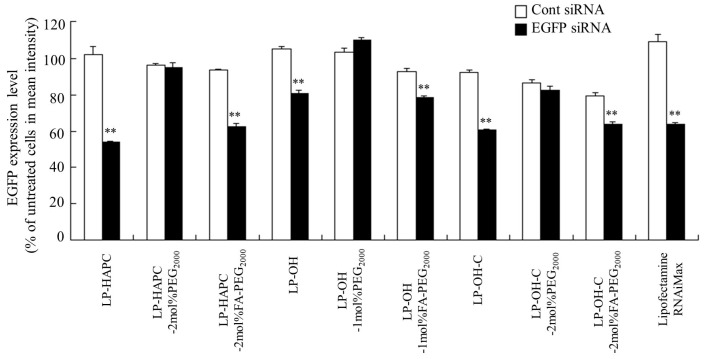
Effect of FA-PEG-modification of cationic liposomes on suppression of EGFP expression in KB-EGFP cells after transfection with FA-PEG-modified siRNA lipoplexes. As cationic liposomes, LP-HAPC, LP-HAPC-2mol%PEG_2000_, LP-HAPC-2mol%FA-PEG_2000_, LP-OH, LP-OH-1mol%PEG_2000_, LP-OH-1mol%FA-PEG_2000_, LP-OH-C, LP-OH-C-2mol%PEG_2000_, and LP-OH-C-2mol%FA-PEG_2000_ were used. The siRNA lipoplexes with Cont siRNA or EGFP siRNA were added to KB-Luc cells at 50 nM siRNA, and the EGFP expression levels were determined on the basis of EGFP-fluorescence by flow cytometry. The EGFP expression level (%) was calculated as relative to the fluorescence intensity of untransfected KB-EGFP cells. Each column represents the mean + SD (*n* = 3). Lipofectamine RNAiMax was used as a control. ** *p* < 0.01, compared with Cont siRNA.

LP-HAPC lipoplexes with EGFP siRNA strongly suppressed EGFP expression (~50% knockdown, compared with Cont siRNA); however, LP-OH and LP-OH-C lipoplexes with EGFP siRNA were suppressed moderately (20–30% knockdown, compared with Cont siRNA) (Figure 7). In contrast, LP-HAPC-2mol%PEG_2000_, LP-OH-1mol%PEG_2000_, and LP-OH-C-2mol%PEG_2000_ lipoplexes did not exhibit suppression of EGFP expression in the cells. However, LP-HAPC-2mol%FA-PEG_2000_, LP-OH-1mol%FA-PEG_2000_, and LP-OH-C-2mol%FA-PEG_2000_ lipoplexes restored the gene silencing effect by FA-PEG-modification. Among the FA-PEG modified siRNA lipoplexes, LP-HAPC-2mol%FA-PEG_2000_ lipoplexes strongly suppressed the expression of EGFP in the cells (~40% knockdown, compared with Cont siRNA), similar to Lipofectamine RNAiMax. Although LP-HAPC-2mol%FA-PEG_2000_, LP-OH-1mol%FA-PEG_2000_, and LP-HAPC-2mol%FA-PEG_2000_ lipoplexes exhibited strong gene silencing effects in KB-Luc cells (Figure 2), they showed moderate gene silencing efficacy in KB-EGFP cells (Figure 7). It has been reported that the t_1/2_ of firefly luciferase protein is about 2–3 h [33,34], but those of GFP and EGFP are more than 24 h [35], indicating that EGFP expression could not be suppressed completely by siRNA due to the long t_1/2_ of EGFP. Among the FA-PEG-modified cationic liposomes, LP-HAPC-2mol%FA-PEG_2000_ appeared to be the most effective in FR-mediated gene silencing.

### 3.7. Antiproliferative Activity

**Figure 8:**
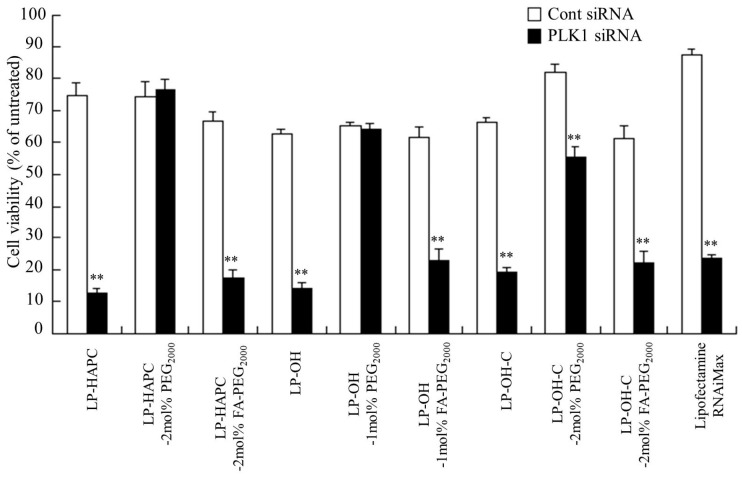
Effect of FA-PEG-modification of cationic liposomes on antiproliferative activities 48 h after transfection with FA-PEG-modified PLK1 siRNA lipoplexes into KB cells. As cationic liposomes, LP-HAPC, LP-HAPC-2mol%PEG_2000_, LP-HAPC-2mol%FA-PEG_2000_, LP-OH, LP-OH-1mol%PEG_2000_, LP-OH-1mol%FA-PEG_2000_, LP-OH-C, LP-OH-C-2mol%PEG_2000_, and LP-OH-C-2mol%FA-PEG_2000_ were used. The siRNA lipoplexes with Cont siRNA or PLK1 siRNA were added to KB-Luc cells at 50 nM siRNA. At 48 h after transfection, cell viability was measured. Each column shows the mean + SD (*n* = 4). Lipofectamine RNAiMax was used as a control. ** *p* < 0.01, compared with Cont siRNA.

PLK1 is a potential target in tumor therapy, because PLK1 is overexpressed in various types of human tumors [3], and its inhibition is potently antiproliferative for tumor cells. To examine whether transfection of PLK1 siRNA into KB cells with FA-PEG modified siRNA lipoplexes could selectivity inhibit tumor growth, we measured cell viability 48 h after transfection of PLK1 siRNA into KB cells. Transfection of LP-HAPC, LP-OH, and LP-OH-C lipoplexes with PLK1 siRNA largely inhibited cell growth; however, LP-HAPC-2mol%PEG_2000_, LP-OH-1mol%PEG_2000_, and LP-OH-C-2mol%PEG_2000_ lipoplexes with PLK1 siRNA showed a decreased cytotoxic effect with the PEG-modification (Figure 8). In contrast, LP-HAPC-2mol%FA-PEG_2000_, LP-OH-1mol%FA-PEG_2000_, and LP-OH-C-2mol%FA-PEG_2000_ lipoplexes with PLK1 siRNA strongly decreased cell proliferation, similar to Lipofectamine RNAiMax.

**Figure 9:**
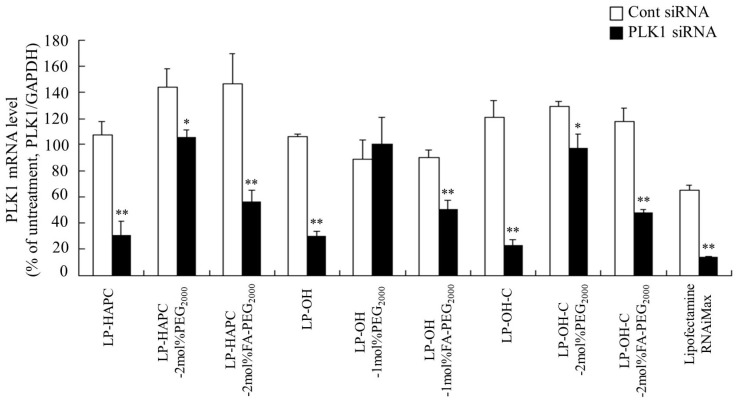
Effect of FA-PEG-modification of cationic liposomes on suppression of PLK1 mRNA expression by transfection with FA-PEG-modified PLK1 siRNA lipoplexes into KB cells. As cationic liposomes, LP-HAPC, LP-HAPC-2mol%PEG_2000_, LP-HAPC-2mol%FA-PEG_2000_, LP-OH, LP-OH-1mol%PEG_2000_, LP-OH-1mol%FA-PEG_2000_, LP-OH-C, LP-OH-C-2mol%PEG_2000_, and LP-OH-C-2mol%FA-PEG_2000_ were used. The siRNA lipoplexes with Cont siRNA or PLK1 siRNA were added to KB-Luc cells at 50 nM siRNA. At 24 h after transfection, the expression levels of PLK1 mRNA in the cells were analyzed by quantitative RT-PCR. Each result represents the mean + SD (*n* = 3). * *p* < 0.05, ** *p* < 0.01, compared with Cont siRNA.

Next, to investigate whether these cytotoxic effects by transfection of PLK1 siRNA were induced by decreased expression level of PLK1 mRNAs, we measured PLK1 mRNA levels at 24 h after transfection of PLK1 siRNA with each cationic liposome. LP-HAPC, LP-OH, and LP-OH-C lipoplexes with PLK1 siRNA significantly inhibited the expression of PLK1 mRNA, compared with Cont siRNA, which was similar to Lipofectamine RNAiMax with PLK1 siRNA (Figure 9). However, LP-HAPC-2mol%PEG_2000_, LP-OH-1mol%PEG_2000_, and LP-OH-C-2mol%PEG_2000_ lipoplexes with PLK1 siRNA did not greatly suppress the expression of PLK1 mRNA. In contrast, transfection of LP-HAPC-2mol%FA-PEG_2000_, LP-OH-1mol%FA-PEG_2000_, or LP-OH-C-2mol%FA-PEG_2000_ siRNA lipoplexes with PLK1 siRNA markedly decreased the expression of PLK1 mRNA in the cells. These results indicated that PLK1 siRNA transfected by FA-PEG-modified lipoplexes could specifically suppress the expression of PLK1 mRNA in the cells, and that suppression of PLK1 mRNA did affect the decrease in proliferation.

### 3.8. In Vivo Gene Therapy in KB Tumor Xenografts

**Figure 10:**
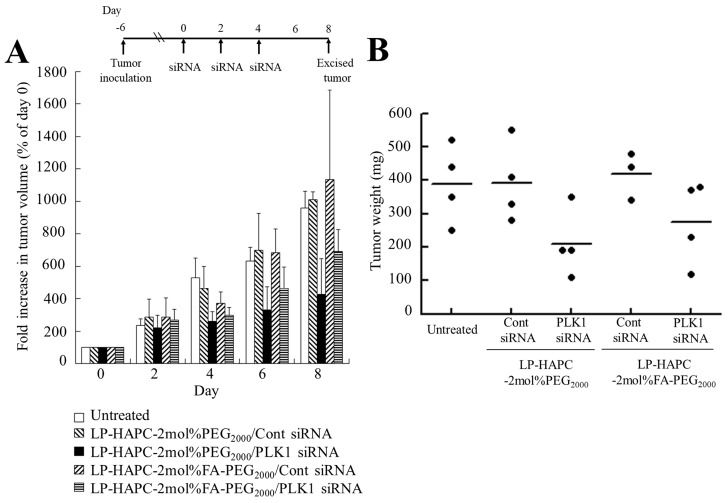
In vivo siRNA therapy of KB tumor xenografts with FA-PEG-modified PLK1 siRNA lipoplexes in mice. When the average volume of the xenograft tumors reached 80 mm^3^ (day 0), LP-HAPC-2mol%PEG_2000_ and LP-HAPC-2mol%FA-PEG_2000_ lipoplexes with 10 μg of Cont siRNA or PLK1 siRNA were injected directly into the tumor three times (day 0, 2, and 4). Tumor volume was measured at days 0, 2, 4, 6, and 8 (**A**). Tumor volume (%) was calculated as relative to each tumor volume at the day 0. The mice were sacrificed at day 8, and then the excised tumors were weighed (**B**). Each result represents the mean + SD (*n* = 3–4).

Among the FA-PEG-modified siRNA lipoplexes, LP-HAPC-2mol%FA-PEG_2000_ lipoplexes strongly suppressed the gene silencing effect in KB cells (Figure 2A and Figure 7). Therefore, we evaluated the anti-tumor effect by direct injection into KB tumor xenografts with LP-HAPC-2mol%FA-PEG_2000_ lipoplexes of PLK1 siRNA. Injection of LP-HAPC-2mol%FA-PEG_2000_ lipoplexes with Cont siRNA or PLK1 siRNA was performed a total of three times, with 2 days between each injection. The anti-tumor effect on KB tumor xenografts was evaluated by measurement of tumor volume (mm^3^) and weight (mg) (Figure 10A,B). Intratumoral injections of LP-HAPC-2mol%FA-PEG_2000_ lipoplexes with PLK1 siRNA inhibited tumor growth compared with the Cont siRNA, although the difference was not significant. However, intratumoral injections of LP-HAPC-2mol%PEG_2000_ lipoplexes with PLK1 siRNA also inhibited growth compared with Cont siRNA, indicating that the in vivo anti-tumor effect by PEG-modified siRNA lipoplexes was not correlated with the in vitro one (Figure 8). This result suggested that the optimal formulation of PEG- or FA-PEG-modified cationic liposomes in FR-selective gene silencing effect might be different between in vitro and in vivo transfection studies.

## 4. Discussion

Several studies have investigated the application of FA-PEG-modified cationic liposomes for siRNA delivery into FR-expressing cells [20,21,22,23], although, many studies have reported on the use of FA-PEG-modified liposomes for the delivery of anticancer drugs and plasmid DNA [36,37]. For siRNA delivery with FA-PEG-modified cationic liposomes, cationic lipids such as dioctadecyldimethylammonium chloride (DODAC) [20,21], 1,2-dioleoyl-3-trimethylammonium-propane (DOTAP) [23], and 3*β*-(*N*-(*N*′,*N*′-dimethylaminoethane)-carbamoyl)cholesterol (DC-Chol) [22] have been used, and FA-PEG-lipids were included at 0.5–2.5 mol% in the liposomal formulations. Previously, we reported that inclusion of 1 mol% FA-PEG_2000_-DSPE into lipid-based cationic nanoparticles composed of OH-Chol and Tween80 could enhance siRNA delivery into tumor cells [30]. However, there have been no reports about the effect of cationic lipid type in FA-PEG-modified cationic liposomes on FR-targeting. Therefore, in this study, we used three types of cationic cholesterol derivatives for the preparation of FA-PEG-modified siRNA lipoplexes, and we examined the effect of cationic lipid type in FA-PEG-modified cationic liposomes on FR-mediated siRNA transfection in tumor cells.

Cationic cholesterol derivatives contain the following parts: a cholesteryl skeleton, a cationic head group, and a linker bound between the cholesteryl skeleton and the cationic head group. The linker between the hydrophilic and hydrophobic parts influences the gene delivery efficacy of cationic liposomes [38]. Furthermore, the introduction of a hydroxyethyl group into the cationic head group of cationic cholesterol derivatives can enhance in vitro gene transfection by cationic liposomes [39,40,41]. Previously, we synthesized HAPC-Chol, OH-Chol, and OH-C-Chol as cationic cholesterol derivatives with a hydroxyethyl group in the cationic head group, and demonstrated that cationic liposomes composed of their cationic cholesterol derivatives exhibited effective siRNA transfection activity [12,13,14,42]. Therefore, in this study, we used their cationic cholesterol derivatives, and prepared three types of FA-PEG-modified siRNA lipoplexes for the evaluation of FR-mediated siRNA transfection.

In in vitro transfection studies, FA-PEG-modification of LP-HAPC, LP-OH, and LP-OH-C could increase the gene silencing effect of siRNAs via efficient cellular uptake, compared with PEG-modification (Figure 2 and Figure 4). In OH-Chol-based cationic liposomes, the increase in FA-PEG_2000_-DSPE in LP-OH-FA-PEG_2000_ increased the amount of siRNA taken up by the cells (Figure 4B). However, it also decreased the gene silencing activity (Figure 2C), indicating that increased FA-PEG-modification of LP-OH could increase cellular uptake of the siRNA lipoplexes via FR, but siRNA might not be efficiently delivered into the cytoplasm or not easily released from the siRNA lipoplexes after FR-mediated endocytosis. In LP-OH-C lipoplexes, the increase in FA-PEG_2000_-DSPE in LP-OH-C-FA-PEG_2000_ decreased the cellular uptake of the siRNA lipoplexes (Figure 4C), but it did not greatly decrease the gene silencing activity (Figure 2D), indicating that the LP-OH-C-1–3mol%FA-PEG_2000_ might deliver siRNA efficiently into cytoplasm after the endocytosis, although the increase of FA-PEG-modification in LP-OH-C decreased the cellular association. In contrast, the increase in FA-PEG_2000_-DSPE in LP-HAPC-FA-PEG_2000_ did not greatly affect the amount of siRNA taken up by the cells (Figure 4A) and the gene silencing effect (Figure 2A), suggesting that FA-PEG-modification of LP-HAPC could mediate cellular uptake via FR, and deliver siRNA efficiently into cytoplasm after the endocytosis. These results indicated that the cellular association via FR and gene silencing efficiency by FA-PEG-modified siRNA lipoplexes were strongly affected by the cationic lipid type in cationic liposomes, and HAPC-Chol will be a better cationic lipid in FR-mediated transfection by cationic liposomes, compared with OH-Chol and OH-C-Chol. However, it was not clear why the cationic lipid type in FA-PEG-modified cationic liposomes affected cellular association via FR and the gene silencing effect. The linker groups of cationic cholesterol derivatives control the flexibility of the cationic head groups. HAPC-Chol and OH-C-Chol have a carbamate-type linker, and OH-Chol has a carboxamide-type linker. Previously, we reported that the difference in the linker group between carboxamide and carbamate in cationic cholesterol derivatives affected cellular association with siRNA nanoplexes [42]. In addition, HAPC-Chol has a slightly longer linker compared with OH-Chol and OH-C-Chol. Therefore, we speculated that the difference in the linker group of these cationic cholesterol derivatives might affect the interaction between the cationic head group on cationic liposomes and the anionic cell membrane and/or endosomal escape after transfection with FA-PEG-modified siRNA lipoplexes.

In our study, the inclusion of PEG_2000_-DSPE or FA-PEG_2000_-DSPE into the formulations of LP-HAPC, LP-OH, and LP-OH-C were optimal at 2 mol%, 1 mol%, and 2 mol%, respectively, for selective FR-mediated gene silencing (Figure 2 and Figure 7). In addition, LP-HAPC-2mol%FA-PEG_2000_, LP-OH-1mol%FA-PEG_2000_, and LP-OH-C-2mol%FA-PEG_2000_ lipoplexes with PLK1 siRNA selectively suppressed the growth of tumor cells via down-regulation of PLK1 mRNA (Figure 8 and Figure 9), indicating that their FA-PEG-modified siRNA lipoplexes were selectively taken up by the FR-expressing cells, and then suppressed the expression of target genes in the cells. However, in in vivo transfection, both LP-HAPC-2mol%PEG_2000_ and LP-HAPC-2mol%FA-PEG_2000_ lipoplexes with PLK1 siRNA inhibited tumor growth compared with Cont siRNA, indicating that the in vivo anti-tumor effect of PEG-modified siRNA lipoplexes was not correlated with the in vitro one. We speculated that siRNA lipoplexes after intratumoral injection were subjected to a strict environment surrounded by tumor cells, resulting in induction of non-specific uptake into tumor cells. Therefore, in in vivo transfection, PEG-modification of LP-HAPC with more PEG_2000_-DSPE might be needed for suppression of the anti-tumor effect by PEG-modified lipoplexes with PLK1 siRNA. However, we reported previously that inclusion of 1 mol% FA-PEG_2000_-DSPE into lipid-based cationic nanoparticles composed of OH-Chol and Tween 80 could efficiently deliver siRNAs into KB cells, and FA-PEG-modified nanoplexes of HER-2 siRNA introduced by intratumoral injection significantly inhibited the tumor growth of KB xenografts compared with Cont siRNA, but PEG-modified nanoplexes did not [30], indicating that the in vivo anti-tumor effects by PEG-modified and FA-PEG-modified siRNA nanoplexes were well correlated with the in vitro ones. These findings suggested that the FR-selective gene silencing by PEG- and FA-PEG-modified cationic liposomes might also be affected by the liposomal formulation. Further studies must be performed to investigate liposomal formulations to improve FR-selective transfection in vivo. In this study, we selected an intratumoral injection as a route of administration for FA-PEG-modified siRNA lipoplexes; however, in future study, we will need to evaluate anti-tumor effect after intravenous injection of FA-PEG-modified siRNA lipoplexes.

## 5. Conclusions

In this study, we examined the effect of cationic lipids in FA-PEG-modified cationic liposomes on gene-silencing effects in tumor cells by siRNA lipoplexes. FA-PEG-modification of cationic liposomes increased in vitro gene-silencing activity regardless of cationic lipid type in cationic liposomes compared with PEG-modification; however, the cationic lipid type in FA-PEG-modified cationic liposomes affected the optimal amount of PEG_2000_-DSPE or FA-PEG_2000_-DSPE in the liposomal formulation. Furthermore, the in vivo anti-tumor effects by PEG-modified and FA-PEG-modified siRNA lipoplexes were not correlated with their in vitro effect, indicating that the optimized formulations of FA-PEG-modified cationic liposomes by in vitro transfection were not necessarily correlated with ones by in vivo transfection. Therefore, the in vivo optimization of FA-PEG-lipids in the liposomal formulation might be important for successful in vivo FR-mediated delivery of siRNAs by FA-PEG modified cationic liposomes.

## Figures and Tables

**Table 1:** Formulation of FA-modified cationic liposomes.

Liposomes	Formulation (mol%)							
	HAPC-Chol	OH-Chol	OH-C-Chol	DOPE	PEG_2000_-DSPE	FA-PEG_2000_-DSPE	PEG_5000_-DSPE	FA-PEG_5000_-DSPE
LP-HAPC	60	-	-	40	-	-	-	-
LP-HAPC-1mol%PEG_2000_	59.4	-	-	39.6	1	-	-	-
LP-HAPC-1mol%PEG_5000_	59.4	-	-	39.6	-	-	1	-
LP-HAPC-2mol%PEG_2000_	58.8	-	-	39.2	2	-	-	-
LP-HAPC-3mol%PEG_2000_	58.2	-	-	38.8	3	-	-	-
LP-HAPC-1mol%FA-PEG_2000_	59.4	-	-	39.6	-	1	-	-
LP-HAPC-1mol%FA-PEG_5000_	59.4	-	-	39.6	-	-	-	1
LP-HAPC-2mol%FA-PEG_2000_	58.8	-	-	39.2	-	2	-	-
LP-HAPC-3mol%FA-PEG_2000_	58.2	-	-	38.8	-	3	-	-
LP-OH	-	60	-	40	-	-	-	-
LP-OH-1mol%PEG_2000_	-	59.4	-	39.6	1	-	-	-
LP-OH-2mol%PEG_2000_	-	58.8	-	39.2	2	-	-	-
LP-OH-3mol%PEG_2000_	-	58.2	-	38.8	3	-	-	-
LP-OH-1mol%FA-PEG_2000_	-	59.4	-	39.6	-	1	-	-
LP-OH-2mol%FA-PEG_2000_	-	58.8	-	39.2	-	2	-	-
LP-OH-3mol%FA-PEG_2000_	-	58.2	-	38.8	-	3	-	-
LP-OH-C	-	-	60	40	-	-	-	-
LP-OH-C-1mol%PEG_2000_	-	-	59.4	39.6	1	-	-	-
LP-OH-C-2mol%PEG_2000_	-	-	58.8	39.2	2	-	-	-
LP-OH-C-3mol%PEG_2000_	-	-	58.2	38.8	3	-	-	-
LP-OH-C-1mol%FA-PEG_2000_	-	-	59.4	39.6	-	1	-	-
LP-OH-C-2mol%FA-PEG_2000_	-	-	58.8	39.2	-	2	-	-
LP-OH-C-3mol%FA-PEG_2000_	-	-	58.2	38.8	-	3	-	-

**Table 2:** Particle size and ζ-potential of cationic liposomes and siRNA lipoplexes.

Liposome	Liposomes			Lipoplexes ^(b)^		
	Size ^(a)^ (nm)	PDI	ζ-Potential ^(a)^ (mV)	Size ^(a)^ (nm)	PDI	ζ-Potential ^(a)^ (mV)
LP-HAPC	103.3 ± 0.3	0.21 ± 0.01	45.4 ± 2.2	183.6 ± 0.2	0.24 ± 0.01	39.5 ± 0.8
LP-HAPC-1mol%PEG_2000_	98.2 ± 2.5	0.25 ± 0.01	46.8 ± 1.0	185.4 ± 3.0	0.28 ± 0.00	31.5 ± 0.1
LP-HAPC-1mol%PEG_5000_	103.1 ± 1.0	0.24 ± 0.01	39.1 ± 0.5	168.8 ± 1.2	0.24 ± 0.01	23.8 ± 0.8
LP-HAPC-2mol%PEG_2000_	101.3 ± 2.5	0.26 ± 0.00	40.7 ± 1.5	179.4 ± 4.1	0.27 ± 0.00	28.3 ± 0.5
LP-HAPC-3mol%PEG_2000_	97.9 ± 2.5	0.22 ± 0.03	37.6 ± 1.1	172.9 ± 1.2	0.27 ± 0.02	31.0 ± 1.3
LP-HAPC-1mol%FA-PEG_2000_	105.3 ± 0.8	0.23 ± 0.01	45.2 ± 0.5	208.1 ± 5.6	0.26 ± 0.02	32.9 ± 1.2
LP-HAPC-1mol%FA-PEG_5000_	101.6 ± 0.7	0.22 ± 0.00	37.6 ± 1.0	204.5 ± 1.8	0.28 ± 0.01	21.4 ± 1.3
LP-HAPC-2mol%FA-PEG_2000_	97.3 ± 0.1	0.23 ± 0.01	41.5 ± 1.1	172.9 ± 1.2	0.27 ± 0.02	28.3 ± 0.9
LP-HAPC-3mol%FA-PEG_2000_	88.3 ± 0.6	0.23 ± 0.01	35.2 ± 0.4	175.7 ± 1.2	0.25 ± 0.01	29.0 ± 1.4
LP-OH	108.9 ± 1.2	0.21 ± 0.01	46.7 ± 1.7	173.5 ± 3.7	0.22 ± 0.02	45.3 ± 0.5
LP-OH-1mol%PEG_2000_	104.1 ± 8.0	0.31 ± 0.02	39.9 ± 1.6	187.9 ± 0.6	0.27 ± 0.01	38.0 ± 2.2
LP-OH-2mol%PEG_2000_	110.5 ± 4.7	0.25 ± 0.01	46.4 ± 3.2	292.1 ± 1.8	0.27 ± 0.01	29.0 ± 1.0
LP-OH-3mol%PEG_2000_	104.4 ± 3.0	0.21 ± 0.01	44.1 ± 1.0	205.8 ± 5.6	0.28 ± 0.01	24.2 ± 0.8
LP-OH-1mol%FA-PEG_2000_	92.6 ± 1.4	0.17 ± 0.01	46.4 ± 1.7	180.1 ± 2.8	0.24 ± 0.01	35.2 ± 1.4
LP-OH-2mol%FA-PEG_2000_	106.4 ± 1.4	0.21 ± 0.01	39.5 ± 0.2	202.3 ± 9.7	0.24 ± 0.03	20.8 ± 0.3
LP-OH-3mol%FA-PEG_2000_	83.5 ± 1.7	0.19 ± 0.01	34.2 ± 1.7	197.7 ± 1.5	0.28 ± 0.01	28.5 ± 0.6
LP-OH-C	91.4 ± 1.4	0.12 ± 0.02	49.1 ± 2.2	172.4 ± 1.9	0.24 ± 0.00	41.4 ± 2.9
LP-OH-C-1mol%PEG_2000_	100.0 ± 0.6	0.24 ± 0.00	43.5 ± 0.7	220.8 ± 5.4	0.26 ± 0.01	38.9 ± 5.7
LP-OH-C-2mol%PEG_2000_	108.5 ± 2.1	0.24 ± 0.01	47.1 ± 1.9	258.2 ± 9.3	0.26 ± 0.01	38.5 ± 1.0
LP-OH-C-3mol%PEG_2000_	110.9 ± 0.9	0.25 ± 0.01	48.8 ± 0.7	201.5 ± 1.9	0.26 ± 0.00	29.8 ± 1.3
LP-OH-C-1mol%FA-PEG_2000_	103.8 ± 2.4	0.23 ± 0.01	51.5 ± 1.6	245.5 ± 1.2	0.12 ± 0.00	31.6 ± 0.7
LP-OH-C-2mol%FA-PEG_2000_	105.8 ± 2.7	0.22 ± 0.01	43.3 ± 1.6	188.8 ± 1.2	0.26 ± 0.01	29.1 ± 1.1
LP-OH-C-3mol%FA-PEG_2000_	104.7 ± 1.9	0.26 ± 0.01	46.6 ± 1.8	415.4 ± 70.0	0.21 ± 0.03	22.0 ± 0.4

PDI: polydispersity index. ^**(a)**^ in water. ^**(b)**^ charge ratio (+:−) of cationic lipid to siRNA phosphate = 7:1. Each value represents the mean ± SD of three measurements per sample.

## Supplementary Materials

The following are available online at https://www.mdpi.com/1999-4923/11/4/181/s1, Figure S1. Effect of charge ratio (+:−) of siRNA lipoplexes on gene suppression in KB-Luc cells after transfection with siRNA lipoplexes.

## References

[1] Wilson R.C., Doudna J.A. (2013). Molecular mechanisms of RNA interference. Annu. Rev. Biophys..

[2] Singh A., Trivedi P., Jain N.K. (2018). Advances in siRNA delivery in cancer therapy. Artif. Cells Nanomed. Biotechnol..

[3] Takai N., Hamanaka R., Yoshimatsu J., Miyakawa I. (2005). Polo-like kinases (PLKS) and cancer. Oncogene.

[4] Takahashi T., Sano B., Nagata T., Kato H., Sugiyama Y., Kunieda K., Kimura M., Okano Y., Saji S. (2003). Polo-like kinase 1 (PLK1) is overexpressed in primary colorectal cancers. Cancer Sci..

[5] Liu Z., Sun Q., Wang X. (2017). PLK1, a potential target for cancer therapy. Transl. Oncol..

[6] Chen X., Mangala L.S., Rodriguez-Aguayo C., Kong X., Lopez-Berestein G., Sood A.K. (2018). RNA interference-based therapy and its delivery systems. Cancer Metastasis Rev..

[7] Wang J., Lu Z., Wientjes M.G., Au J.L. (2010). Delivery of siRNA therapeutics: Barriers and carriers. AAPS J..

[8] Zhang S., Zhi D., Huang L. (2012). Lipid-based vectors for siRNA delivery. J. Drug Target..

[9] Martin B., Sainlos M., Aissaoui A., Oudrhiri N., Hauchecorne M., Vigneron J.P., Lehn J.M., Lehn P. (2005). The design of cationic lipids for gene delivery. Curr. Pharm. Des..

[10] Xue H.Y., Guo P., Wen W.C., Wong H.L. (2015). Lipid-based nanocarriers for RNA delivery. Curr. Pharm. Des..

[11] Ariatti M. (2015). Liposomal formulation of monovalent cholesteryl cytofectins with acyclic head groups and gene delivery: A systematic review. Curr. Pharm. Biotechnol..

[12] Hattori Y., Takeuchi N., Nakamura M., Yoshiike Y., Taguchi M., Ohno H., Ozaki K., Onishi H. (2018). Effect of cationic lipid type in cationic liposomes for siRNA delivery into the liver by sequential injection of chondroitin sulfate and cationic lipoplex. J. Drug Deliv. Sci. Technol..

[13] Hattori Y., Nakamura M., Takeuchi N., Tamaki K., Shimizu S., Yoshiike Y., Taguchi M., Ohno H., Ozaki K., Onishi H. (2019). Effect of cationic lipid in cationic liposomes on siRNA delivery into the lung by intravenous injection of cationic lipoplex. J. Drug Target..

[14] Hattori Y., Hara E., Shingu Y., Minamiguchi D., Nakamura A., Arai S., Ohno H., Kawano K., Fujii N., Yonemochi E. (2015). SiRNA delivery into tumor cells by cationic cholesterol derivative-based nanoparticles and liposomes. Biol. Pharm. Bull..

[15] Wasungu L., Hoekstra D. (2006). Cationic lipids, lipoplexes and intracellular delivery of genes. J. Control. Release.

[16] Liu F., Huang L. (2002). Development of non-viral vectors for systemic gene delivery. J. Control. Release.

[17] Parker N., Turk M.J., Westrick E., Lewis J.D., Low P.S., Leamon C.P. (2005). Folate receptor expression in carcinomas and normal tissues determined by a quantitative radioligand binding assay. Anal. Biochem..

[18] Low P.S., Kularatne S.A. (2009). Folate-targeted therapeutic and imaging agents for cancer. Curr. Opin. Chem. Biol..

[19] Xia W., Low P.S. (2010). Folate-targeted therapies for cancer. J. Med. Chem..

[20] Lopes I., Oliveira C.N., Sárria M.P., Neves Silva J.P., Goncalves O., Gomes A.C., Real Oliveira M.E. (2016). Monoolein-based nanocarriers for enhanced folate receptor-mediated RNA delivery to cancer cells. J. Liposome Res..

[21] Feng C., Wang T., Tang R., Wang J., Long H., Gao X., Tang S. (2010). Silencing of the MYCN gene by siRNA delivered by folate receptor-targeted liposomes in LA-N-5 cells. Pediatr. Surg. Int..

[22] Xiang B., Dong D.W., Shi N.Q., Gao W., Yang Z.Z., Cui Y., Cao D.Y., Qi X.R. (2013). PSA-responsive and PSMA-mediated multifunctional liposomes for targeted therapy of prostate cancer. Biomaterials.

[23] Yang T., Li B., Qi S., Liu Y., Gai Y., Ye P., Yang G., Zhang W., Zhang P., He X. (2014). Co-delivery of doxorubicin and bmi1 siRNA by folate receptor targeted liposomes exhibits enhanced anti-tumor effects in vitro and in vivo. Theranostics.

[24] Hattori Y., Kubo H., Higashiyama K., Maitani Y. (2005). Folate-linked nanoparticles formed with DNA complexes in sodium chloride solution enhance transfection efficiency. J. Biomed. Nanotechnol..

[25] Takeuchi K., Ishihara M., Kawaura C., Noji M., Furuno T., Nakanishi M. (1996). Effect of zeta potential of cationic liposomes containing cationic cholesterol derivatives on gene transfection. FEBS Lett..

[26] Ding W., Hattori Y., Higashiyama K., Maitani Y. (2008). Hydroxyethylated cationic cholesterol derivatives in liposome vectors promote gene expression in the lung. Int. J. Pharm..

[27] Hattori Y., Kikuchi T., Ozaki K., Onishi H. (2017). Evaluation of in vitro and in vivo therapeutic antitumor efficacy of transduction of polo-like kinase 1 and heat shock transcription factor 1 small interfering RNA. Exp. Ther. Med..

[28] Hattori Y., Arai S., Kikuchi T., Ozaki K., Kawano K., Yonemochi E. (2016). Therapeutic effect for liver-metastasized tumor by sequential intravenous injection of anionic polymer and cationic lipoplex of siRNA. J. Drug Target..

[29] Shiokawa T., Hattori Y., Kawano K., Ohguchi Y., Kawakami H., Toma K., Maitani Y. (2005). Effect of polyethylene glycol linker chain length of folate-linked microemulsions loading aclacinomycin a on targeting ability and antitumor effect in vitro and in vivo. Clin. Cancer Res..

[30] Yoshizawa T., Hattori Y., Hakoshima M., Koga K., Maitani Y. (2008). Folate-linked lipid-based nanoparticles for synthetic siRNA delivery in KB tumor xenografts. Eur. J. Pharm. Biopharm..

[31] Livak K.J., Schmittgen T.D. (2001). Analysis of relative gene expression data using real-time quantitative PCR and the 2^−ΔΔ*C*t^ method. Methods.

[32] Hattori Y., Yamashita J., Sakaida C., Kawano K., Yonemochi E. (2015). Evaluation of antitumor effect of zoledronic acid entrapped in folate-linked liposome for targeting to tumor-associated macrophages. J. Liposome Res..

[33] Ignowski J.M., Schaffer D.V. (2004). Kinetic analysis and modeling of firefly luciferase as a quantitative reporter gene in live mammalian cells. Biotechnol. Bioeng..

[34] Bronstein I., Fortin J., Stanley P.E., Stewart G.S., Kricka L.J. (1994). Chemiluminescent and bioluminescent reporter gene assays. Anal. Biochem..

[35] Corish P., Tyler-Smith C. (1999). Attenuation of green fluorescent protein half-life in mammalian cells. Protein Eng..

[36] Hattori Y., Maitani Y. (2005). Folate-linked lipid-based nanoparticle for targeted gene delivery. Curr. Drug Deliv..

[37] Chaudhury A., Das S. (2015). Folate receptor targeted liposomes encapsulating anti-cancer drugs. Curr. Pharm. Biotechnol..

[38] Rajesh M., Sen J., Srujan M., Mukherjee K., Sreedhar B., Chaudhuri A. (2007). Dramatic influence of the orientation of linker between hydrophilic and hydrophobic lipid moiety in liposomal gene delivery. J. Am. Chem. Soc..

[39] Okayama R., Noji M., Nakanishi M. (1997). Cationic cholesterol with a hydroxyethylamino head group promotes significantly liposome-mediated gene transfection. FEBS Lett..

[40] Percot A., Briane D., Coudert R., Reynier P., Bouchemal N., Lievre N., Hantz E., Salzmann J.L., Cao A. (2004). A hydroxyethylated cholesterol-based cationic lipid for DNA delivery: Effect of conditioning. Int. J. Pharm..

[41] Biswas J., Mishra S.K., Kondaiah P., Bhattacharya S. (2011). Syntheses, transfection efficacy and cell toxicity properties of novel cholesterol-based gemini lipids having hydroxyethyl head group. Org. Biomol. Chem..

[42] Hattori Y., Hagiwara A., Ding W., Maitani Y. (2008). Nacl improves sirna delivery mediated by nanoparticles of hydroxyethylated cationic cholesterol with amido-linker. Bioorg. Med. Chem. Lett..

