# Supplementary material for: Effect of Cationic Lipid Type in Folate-PEG-Modified Cationic Liposomes on Folate Receptor-Mediated siRNA Transfection in Tumor Cells
Source: Pharmaceutics. 2019 Apr 15;11(4):181. doi: 10.3390/pharmaceutics11040181 (PMC6523911; doi:10.3390/pharmaceutics11040181)
Supplement: Supplementary file 1 [file pharmaceutics-11-00181-s001.pdf]

pharmaceutics

Supplementary Materials: Effect of Cationic Lipid Type in Folate-PEG-Modified Cationic Liposomes on Folate Receptor-Mediated siRNA Transfection in Tumor Cells

Yoshiyuki Hattori, Satono Shimizu, Kei-ichi Ozaki and Hiraku Onishi

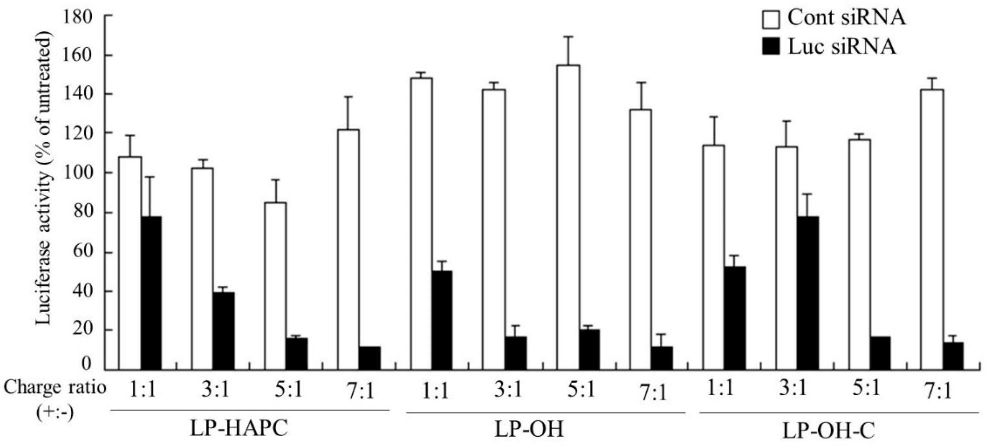

Figure S1. Effect of charge ratio (+:−) of siRNA lipoplexes on gene suppression in KB-Luc cells after transfection with siRNA lipoplexes. siRNA lipoplexes were formed at charge ratios (+:−) of 1:1, 3:1, 5:1, and 7:1. siRNA lipoplexes with Cont siRNA or Luc siRNA were added to KB-Luc cells at 50 nM siRNA, and luciferase assays were carried out after 48 h incubation. Each column represents the mean+S.D. (n = 3).
